# Ethnic, Botanic, Phytochemistry and Pharmacology of the *Acorus* L. Genus: A Review

**DOI:** 10.3390/molecules28207117

**Published:** 2023-10-16

**Authors:** Yu Zhao, Jia Li, Guoshi Cao, Daqing Zhao, Guangzhe Li, Hongyin Zhang, Mingming Yan

**Affiliations:** 1 Northeast Asia Research Institute, Changchun University of Chinese Medicine, Changchun 130117, China; 13089231233@163.com (Y.Z.); m17790057519@163.com (J.L.); 15526829853@163.com (G.C.); zhaodaqing1963@163.com (D.Z.); ligz@nenu.edu.cn (G.L.); 2 Jilin Provincial Science and Technology Innovation Center of Health Food of Chinese Medicine, Changchun University of Chinese Medicine, Changchun 130117, China

**Keywords:** *Acorus*, terpenoids and phenylpropanoids, asarone, neuroprotective, Alzheimer’s disease

## Abstract

The genus *Acorus*, a perennial monocotyledonous-class herb and part of the Acoraceae family, is widely distributed in the temperate and subtropical zones of the Northern and Southern Hemispheres. *Acorus* is rich in biological activities and can be used to treat various diseases of the nervous system, cardiovascular system, and digestive system, including Alzheimer’s disease, depression, epilepsy, hyperlipidemia, and indigestion. Recently, it has been widely used to improve eutrophic water and control heavy-metal-polluted water. Thus far, only three species of *Acorus* have been reported in terms of chemical components and pharmacological activities. Previously published reviews have not further distinguished or comprehensively expounded the chemical components and pharmacological activities of *Acorus* plants. By carrying out a literature search, we collected documents closely related to *Acorus* published from 1956 to 2022. We then performed a comprehensive and systematic review of the genus *Acorus* from different perspectives, including botanical aspects, ethnic applications, phytochemistry aspects, and pharmacological aspects. Our aim was to provide a basis for further research and the development of new concepts.

## 1. Introduction

The genus *Acorus* L. (Acoraceae), a perennial monocotyledonous-class herb belonging to the Acoraceae family, is extensively distributed throughout the temperate and subtropical zones of the Northern and Southern Hemispheres, including India, Kazakhstan, China, and Japan [1,2]. *Acorus* has been used as a traditional medicinal plant for the treatment of various diseases for over 2000 years [3]. *Acorus* is termed “Chang Pu” in Chinese; it was first recorded in Shennong’s Material Medicine source as a first-class variety used to treat epilepsy, palpitations, abdominal pain, bruising, forgetfulness, etc. [4,5]. Research on the chemical and pharmacological activities of *Acorus* has mainly focused on three species: *A. calamus*, *A. tatarinowii*, and *A. gramineus* [6]. *A. calamus* (and varieties) and *A. gramineus* are listed in the Plant List and International Plant Names Index. *A. calamus* and *A. tatarinowii* are listed in the Chinese Pharmacopoeia. *Acorus* plants have a fragrant odor. The volatile oil of *Acorus* is considered to be its main active ingredient. It is used for medicine and also as a spice. *Acorus* contains various chemical components, including terpenoids, phenylpropanoids, flavonoids, and alkaloids. Modern pharmacology has indicated that these components have various pharmacological activities and that they can be used to treat diseases of the nervous system, cardiovascular system, and digestive system, including Alzheimer’s disease, depression, anxiety, hyperglycemia, hyperlipidemia, gastric colic, and indigestion [7,8].

*Acorus* plants not only have an extensive edible and medicinal value but also have an important ecological and environmental value; they are widely used in the treatment of polluted water bodies. As an important plant in artificial wetlands, *Acorus* can restore eutrophic water bodies and absorb pollutants such as N and P, along with heavy metals in the water; they are the primary selected plant when treating heavy-metal-polluted water bodies [9,10,11].

Despite recent interest in investigating the rich pharmacological effects and clinical application value of *Acorus*, there have been no comprehensive reviews in the last decade of its other aspects. Using the keywords “*Acorus*”, “Chang Pu”, “Acoraceae”, “Phytochemistry”, and “Pharmacology”, we compiled the relevant literature from scientific databases such as Web of Science, PubMed, Google Scholar, ACS Publications, ScienceDirect, and CNKI. Domestic and international academic research on the chemical composition and pharmacological activity of *Acorus* provides a theoretical foundation for the national pharmacological application of *Acorus*. In this paper, we document the relevant research on the botany, chemistry, pharmacology, and environmental protection of *Acorus* from 1956 to 2022. Our objective was to meticulously and exhaustively assess the medicinal and environmental protection potential of *Acorus* based on the available data; we hope that this review will serve as a constructive guide for future applications and developments.

## 2. Ethnic Application

In China, *Acorus* has been used for sustenance, medicine, and as an indoor bonsai for over two thousand years. *Acorus* plants are one of the most frequently used medicinal botanicals among Chinese ethnic minorities. According to the available statistics, 29 ethnic minorities in China use *A. calamus*, *A. tatarinowii*, and *A. gramineus* to alleviate diseases. For instance, the Miao nationality mainly uses *A. tatarinowii* to treat cardiovascular disease, *A. calamus* to treat dysentery, and *A. gramineus* to treat chest distentions, nausea, and loss of appetite. The Bouyei nationality uses *A. calamus* to treat appendicitis. The Huaqiao nationality uses *A. calamus* to treat eye swelling, irregular menstruation, etc. The Hani people use *A. calamus* to treat chronic tracheitis, anorexia, and enteritis; *A. tatarinowii* to soothe the nerves, promote blood circulation, regulate the stomach, and dispel dampness; and *A. gramineus* to treat rheumatoid arthritis and dyspepsia. The Deang nationality uses *A. calamus* to treat abdominal pain. The Mulam people collect *Acorus* flowers to treat wounds, among other purposes [12,13]. *Acorus* plants can also be used with other herbs to treat diseases. 

*Acorus* is extensively used not only in China but also in India, Korea, and other nations around the world. Since ancient times, India has used the roots and leaves of *A. calamus* as a repellent and to treat various diseases. *A. calamus* has been used in the Indian Ayurvedic tradition of medicine for analgesic, anti-pyretic, tonic, anti-obesity, and healing purposes. The rhizome of *A. calamus* has been used to rectify speech defects and improve the memory of children in rural areas of southern India. It has also been used in the treatment of migraines, earache, and tinnitus. Its nasal administration is beneficial for headaches and epilepsy [14,15,16,17]. In the United States and Indonesia, *A. calamus* has been extensively used in traditional folk medicine for the treatment of diabetes [18]. In Nepal, *A. calamus* has been used to lower blood pressure and treat coughs, headaches, and snake bites [19,20]. In Korea and India, *Acorus* has been enumerated in the pharmacopoeia due to its various pharmacological activities. It has been used for centuries due to its positive effects on learning and memory [21]. In Asia, Europe, and North America, *A. calamus* has been viewed as a folk remedy to treat arthritis, neuralgia, diarrhea, dyspepsia, alopecia, and other conditions [22,23]. Many Native American tribes in the United States use *A. calamus* as an anesthetic for headaches and toothache; thus, *A. calamus* may be a potential anesthetic [24]. From the perspective of modern pharmacology, the national medicinal use of *Acorus* primarily manifests in the treatment of diseases that affect the nervous system, digestive system, cardiovascular system, and other diseases, including the alleviation of headaches, the treatment of epilepsy, improvements in diabetes and obesity, and the treatment of diarrhea and indigestion. Modern pharmacological experiments have confirmed these traditional pharmacological effects, which are progressively becoming safe and credible.

*A. gramineus* and *A. tatarinowii* are commonly used as flavoring agents for food such as stewed meat and fish to remove odors and enhance flavor. Its rhizome contains volatile oil that can be extracted for flavoring purposes [2]. Since the Han Dynasty, *A. tatarinowii* has been used to produce wine; it promotes blood circulation and improves eyesight. *A. calamus* oil is used as a flavoring agent, especially in alcoholic beverages and ales. In Europe, the rhizomes and essential oil of *A. calamus* are extensively used in the flavoring industry and in the manufacturing of alcoholic beverages. The essence extracted from the *A. calamus* rhizome is used to flavor pipe tobacco. It is also employed in the manufacturing of toothpastes and fragrances [24]. Many modern pharmacological experiments have also confirmed these traditional pharmacological effects, and the medicinal use of *A. calamus* is gradually becoming safe and reliable.

In addition to its medicinal value, *Acorus* has also been endowed with a strong cultural connotation; it is one of the representative elements of China’s Dragon Boat Festival. *Acorus* plants are essential for all ethnic minorities participating in the Dragon Boat Festival. The local populace considers *Acorus* to be a sacred plant. To exorcise evil and pray for peace, people hang *Acorus* and wormwood together on doors, create *Acorus* sachets to wear, use *Acorus* as a herbal bathing material, and consume *Acorus* wine. In ancient China, *Acorus* was combined with orchids, narcissi, and chrysanthemums to form the four most elegant blossoms and plants. It is also used as an indoor bonsai due to both its ornamental and anti-air pollution effects and for the adsorption of fine dust in the air [25].

## 3. Botany

According to the biological classification system of Swedish biologist Linnaeus [26,27], *Acorus* is placed in the Acoraceae family in the monocotyledonous class of the Arales order and *Angiospermae* phylum. Thus far, there are approximately nine varieties in the world: *Acorus calamus* L., *Acorus tatarinowii* Schott, *Acorus rumphianus* S. Y. Hu, *Acorus gramineus* Soland, *Acorus macrospadiceus* F. N. Wei, *Acorus xingyeus* Z. Y. Zhu, *Acorus latifolius* Z. Y. Zhu, *Acorus calamus* L. var. Verus L., and *Acorus tatarinowii* Schott var. *flavo-marginatus* K. M. Liu [28,29].

*Acorus* plants are semi-evergreen, rhizomatous perennials that form tufts of linear or sword-shaped leaves and spike-like flowers near the leaf-like extremities of its central stems. They are 5 to 6 cm long and have fleshy, aromatic, and fibrous roots. The leaves are basal, with membranous leaf sheaths on the basal sides that are 4–5 mm wide, tapering upwards to one-third of the leaf length and progressively falling off. The leaf blade is linear and sword-shaped, 90–100 cm long, and 1–2 cm wide at the middle; it is broad and conduplicate at the base and tapers above the middle. The leaves are herbaceous, green, and shiny; the midrib is conspicuously elevated on both surfaces. There are three to five pairs of lateral veins, which are parallel, slender, and primarily extend to the leaf tip. The inflorescence stalk is triangular and 40–50 cm in length. The spathe is linear and sword-shaped; it is 30–40 cm in length [1,29].

## 4. Chemistry

**Figure 1:**
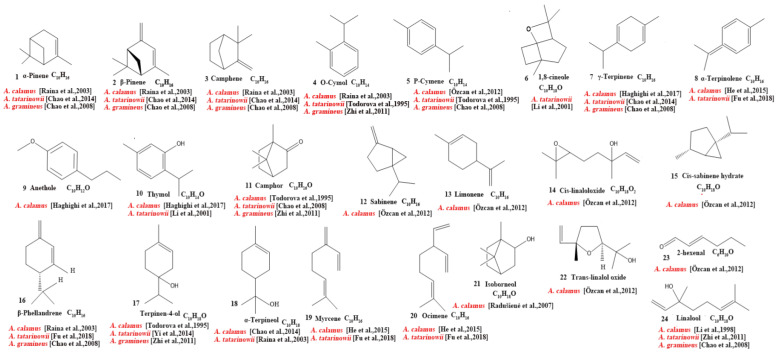
Chemical structures of monoterpenes from the *Acorus* genus [22,32,33,35,36,37,38,39,40,41,43].

**Figure 2:**
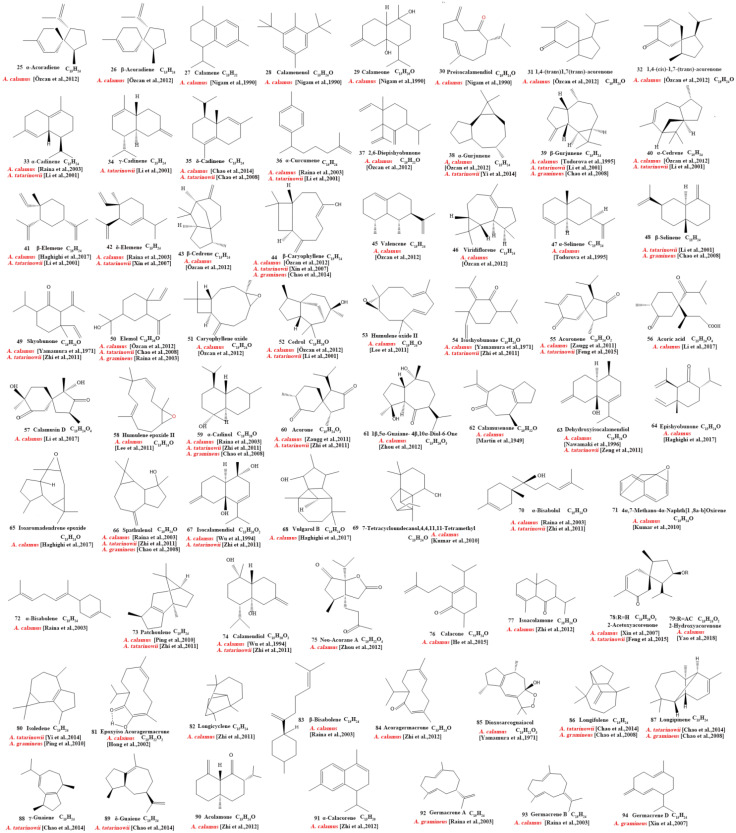
Chemical structures of sesquiterpenes from the *Acorus* genus [22,32,33,34,35,36,37,38,42,44,45,46,47,48,49,50,51,52,53,54,55,56,57,58,59,60].

**Figure 3:**
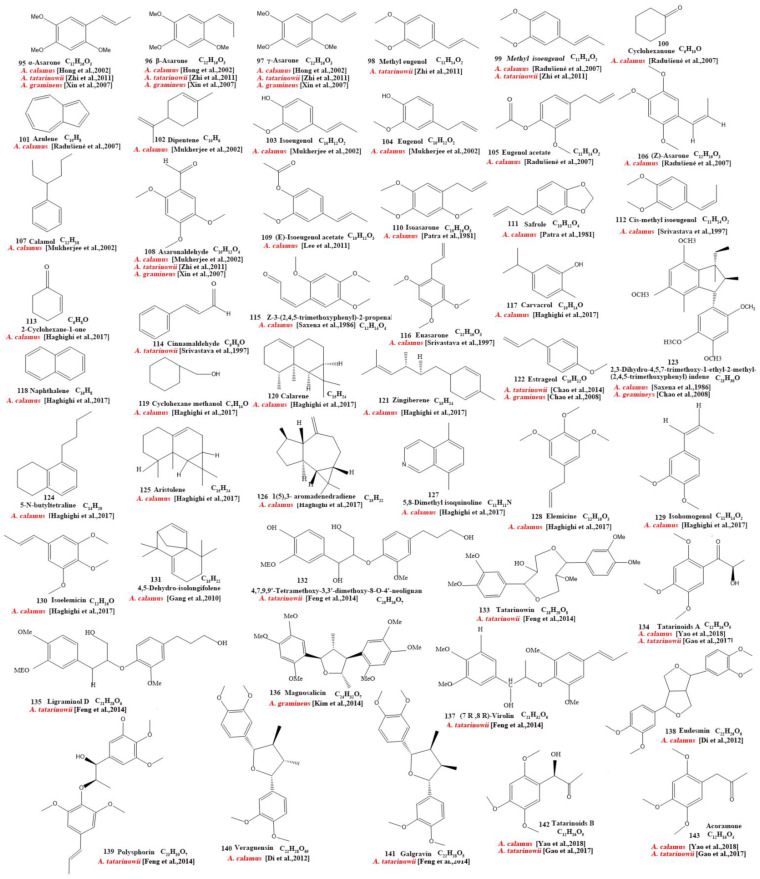

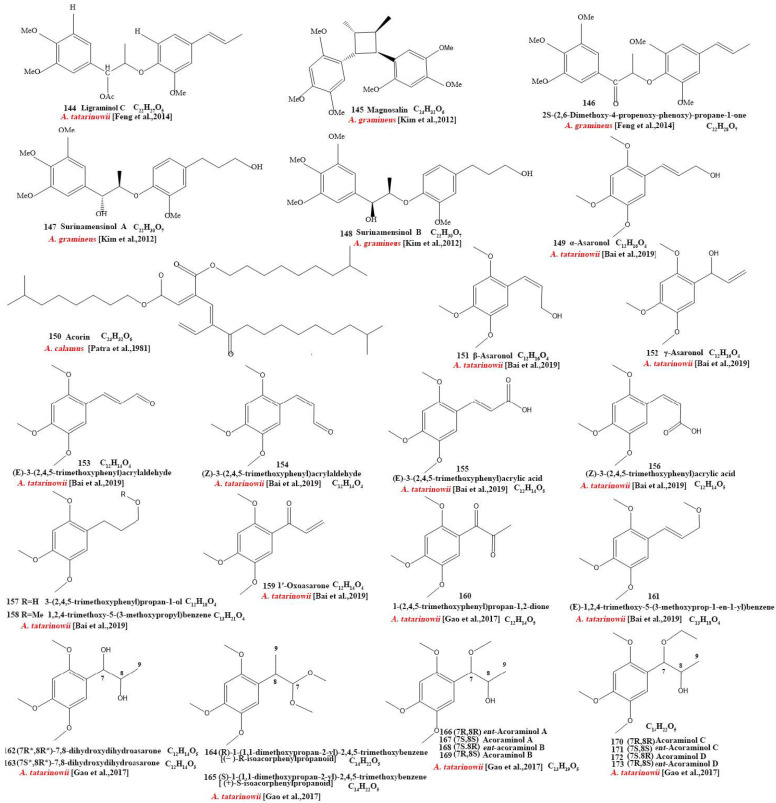
Chemical structures of phenylpropanoid from the *Acorus* genus [32,33,35,38,43,45,47,59,61,62,63,64,65,66,67,68,70,71,81].

Our review of the literature revealed that 224 compounds have been isolated and identified from *Acorus*. These include monoterpenes ((1–24) [22,30,31,32,33,34,35,36,37,38,39,40,41], sesquiterpenes (25–94) [22,30,31,32,33,34,35,36,37,40,42,43,44,45,46,47,48,49,50,51,52,53,54,55,56,57,58], phenylpropanoids (95–173) [30,31,33,36,41,43,45,59,60,61,62,63,64,65,66,67,68,69,70], alkaloids (174–194) [46,48,54,57,64,69,70,71], flavonoids (195–204) [37,61,69,72,73], and other compounds (205–224) [37,73,74,75]. The main pharmacologically active ingredient of *Acorus* is volatile oil; its oil content varies depending on its source and place of origin and ranges from 0.5% to 3.27%. Terpenoids and phenylpropanoids compounds are the main components of the volatile oil in *Acorus*. To date, 24 monoterpenoids (Figure 1) and 70 sesquiterpenoids (Figure 2) have been found in *Acorus*, all of which have relatively simple structures and are common monoterpenoids. These monoterpenes ingredients can promote wound healing and wound contractions; their increased tensile strength and increased hydroxyproline content support the further evaluation of *Acorus* in the topical treatment of wounds as well as its anti-inflammatory effect [76]. As sesquiterpenoid compounds, Neoacorane A, aconitic acid, and Calamusin D are considered to be potential nerve cell-protective leads, but further research evidence on this prospect is required [50]. A total of 79 phenylpropanoid compounds have been identified in the *Acorus* genus (Figure 3). The composition of asarone, three isomers characterized by the presence of a 1,2,4-trimethoxybenzene moiety, has been extensively studied. α-Asarone and β-asarone have various pharmacological properties, including anti-epilepsy, neuroprotective, and lipid-lowering properties; they have also been used as a treatment for Alzheimer’s disease. There is limited research on γ-asarone; further research is required regarding its pharmacological effects. α-Asarone and β-asarone are isomers of each other; pharmacokinetic studies have observed that approximately 22% β-asarone can be converted into α-asarone [77]. α-Asarone and β-asarone are widely distributed in the brain, indicating that they can penetrate the blood–brain barrier; thus, they have the potential to treat central nervous system diseases. Methyl eugenol ether is a relatively high-quality phenylpropanoid component in *Acorus* (20.81%). It exudes a fragrance similar to lilacs and carnations. It is a food-grade spice that has been approved by the state and can be used as a baking essence and tobacco essence.

**Figure 4:**
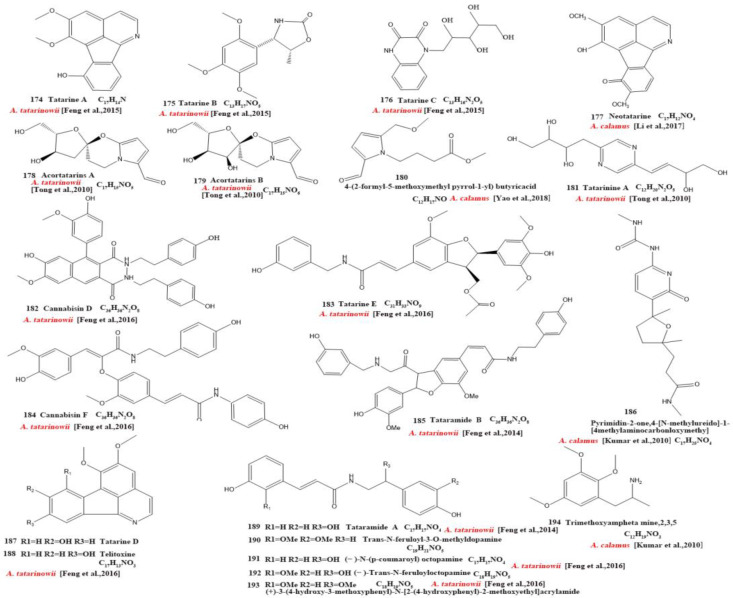
Chemical structures of alkaloids from the *Acorus* genus [49,50,56,59,68,72,73,78].

**Figure 5:**
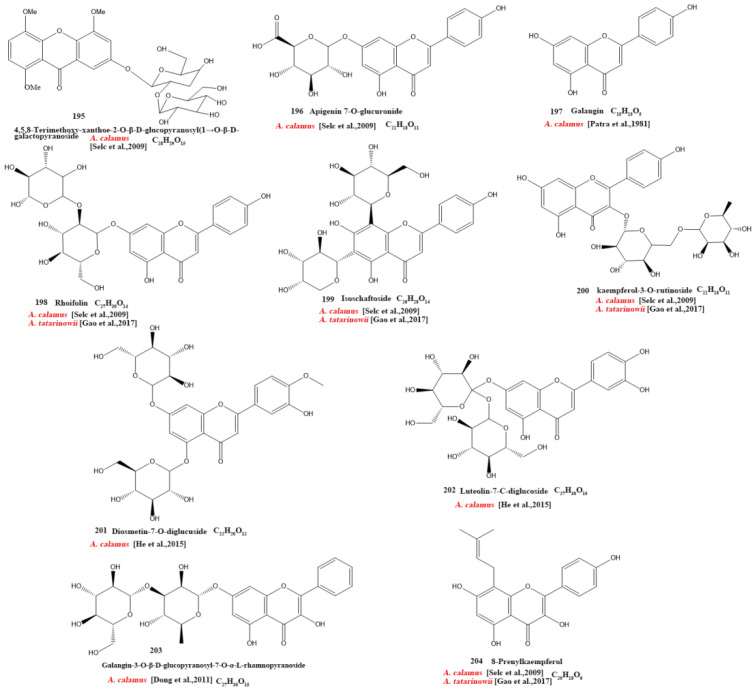
Chemical structures of flavonoids from the *Acorus* genus [39,63,71,74,75].

**Figure 6:**
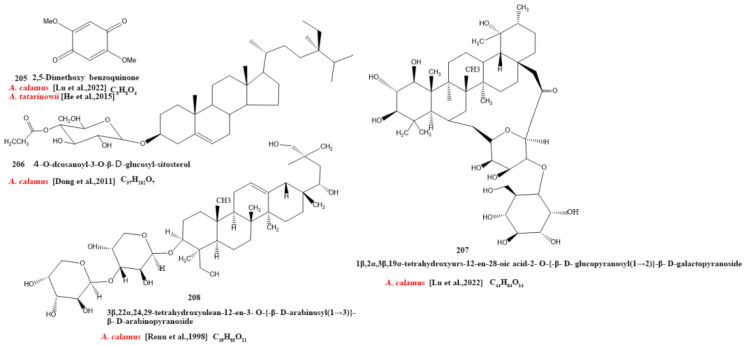
Chemical structures of other ingredients from the *Acorus* genus [39,75,76,77].

Currently, 21 alkaloids (174–194) have been identified and isolated from *Acorus* (Figure 4). These azafluoranthene alkaloids are a class of unique-structured compounds with memory-enhancing pharmacological and anti-dementia activities and the potential to be used for the treatment of Alzheimer’s disease [78]. Neotatarine, a novel alkaloid, has been isolated from the rhizome of *A. calamus* and shown to exhibit neuroprotective properties against A25–35-induced neurotoxicity in PC12 cells. The structure of neotatarine is derived from 7*H*-azuleno [1,2,3-i,j] isoquinolin-7-one. It is currently the only compound observed in nature with a 7*H*-azuleno [1,2,3-i,j] isoquinolin-7-one structural mother nucleus [79]. In total, 10 flavonoids (195–204) have been isolated from and identified in *Acorus* (Figure 5); the majority are flavonoid glycosides with a quinone compound (205) and three triterpenoid saponins (206–208) (Figure 6). *Acorus* also contains amino acids, fatty acids, inorganic elements, etc. To date, scientists have identified 12 amino acid components (Tryptophan, Aspartic acid, Phenylalanine, Lysine, Norvaline, α-alanine, Arginine, Asparagine, Threonine, Proline, Tyrosine, Glutamic acid) and 4 fatty acid components from (Palmitic acid, Myristic acid, Palmitoleic acid, Stearic acid) *A. calamus and A. tatarinowii*, as well as inorganic elements such as potassium, sodium, calcium, magnesium, manganese, and iron [6,80].

## 5. Pharmacology

**Figure 7:**
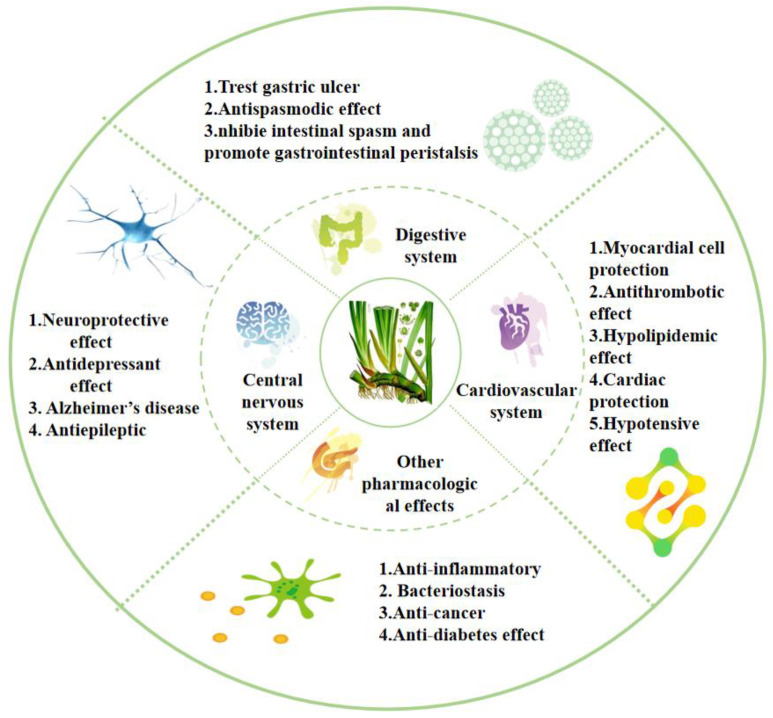
Pharmacological activities of the *Acorus* genus.

*Acorus* has a long medicinal history. The most commonly used parts are the rhizomes, which are widely used to treat various ailments. The whole plant, as well as leaves and inflorescences, is used in medicines [1]. Numerous in vivo and in vitro experimental studies have demonstrated that *Acorus* has extensive pharmacological effects (Figure 7); for example, treating diseases such as Alzheimer’s disease, depression, epilepsy, hypertension, hyperlipidemia, thrombosis, indigestion, gastric colic, and diarrhea. It also has pharmacological effects, including anti-bacterial, anti-tumor, and anti-inflammatory properties. It can be used for the treatment of multiple systemic diseases, such as those affecting the nervous system, cardiovascular system, and digestive system. Therefore, the further exploration and development of its pharmacological mechanism are of significance.

### 5.1. Effect on Central Nervous System

#### 5.1.1. Neuroprotective Activity

**Figure 8:**
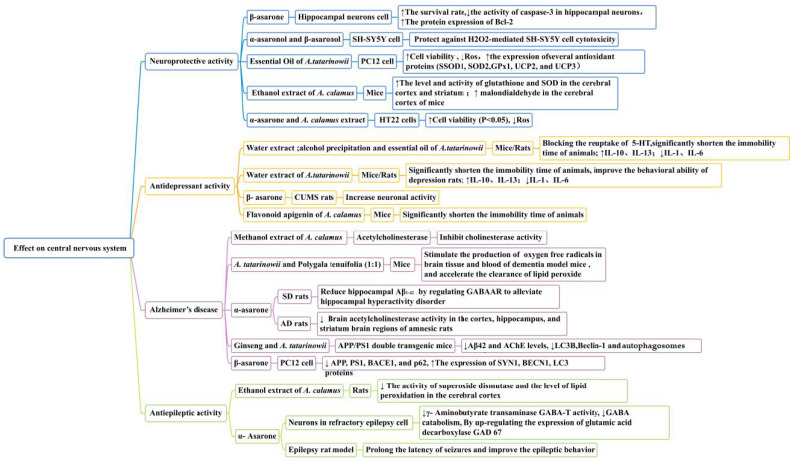
Effect on central nervous system of the *Acorus* genus.

*Acorus* plants are excellent drugs for the treatment of neurological diseases (Figure 8). *Acorus* volatile oil components are the main active compounds with sedative and neuroprotective effects. In vitro research has revealed that α-asaronol and β-asaronol could protect SH-SY5Y cells from H_2_O_2_-mediated cytotoxicity. In one study, at doses between 0.01 and 100 M, α-asaronol and β-asaronol demonstrated potential neuroprotective effects against neuronal oxidative stress, with β-asaronol having the strongest effect [70]. 

Research has revealed that β-asarone (3.6 × 10^−5^ mol/L, 7.2 × 10^−5^ mol/L, 14.4 × 10^−5^ mol/L, 28.8 × 10^−5^ mol/L, and 57.6 × 10^−5^ mol/L) could significantly increase the survival rate of hippocampal neuron cells injured by glucose deficiency and hypoxia reperfusion (*p* < 0.05), with a time-dependent relationship (but not a dose-dependent relationship) between 6 and 24 h. β-Asarone has also been shown to decrease the activity of caspase-3 in hippocampal neurons (*p* < 0.05), inhibiting their apoptosis in [82]. By inhibiting the JNK-mediated mitochondrial pathway, neurons were protected after a mild cerebral ischemia reperfusion. On the basis of these results, we believe that β-asarone may be used to treat cerebral ischemia reperfusion injuries.

In another study, the essential oil of *A. tatarinowii* (at 1.5, 5, and 15 μg/mL) prevented hydrogen-peroxide-induced cell injury in PC12 cells, increased the viability of cells affected by H_2_O_2_-mediated injury, inhibited reactive oxygen species (ROS) accumulation, and induced the expression of several anti-oxidant proteins (SSOD1, SOD2, GPx1, UCP2, and UCP3). A maximal induction increase of >100% was observed at 15 μg/mL. The underlying mechanism of this action may be related to the upregulatory effect on CREB/PGC-1α signaling [83]. 

Research has revealed that plant extracts and preparations with anti-oxidant properties have neuroprotective effects in ischemic brain injuries. An extract of *A. calamus* may exert neuroprotective effects through anti-oxidant effects. The ethanol extract of *A. calamus* (25 mg/kg) significantly reduced the level of malondialdehyde in the cerebral cortex of mice with cerebral artery occlusions (*p* < 0.05); the level and activity of glutathione and SOD in the cerebral cortex and striatum were significantly increased (*p* < 0.01 and *p* < 0.01, respectively). This indicated that the ethanol extract of *A. calamus* had a neuroprotective effect on this cerebral ischemia model of mice [84]. *A. calamus* extract (20 and 200 μg/mL) and α-asarone (0.5 and 50 μm) both significantly suppressed HT22 cell death induced by the oxidative stress inducer L-glutamate and the ER stress inducer tunicamycin. They also significantly protected hippocampal cells against oxidative stress and ER stress by decreasing ROS production and suppressing PERK signaling [85]. At a molecular level, this neuroprotection could be attributed to the anti-oxidant and anti-apoptotic effects of *A. calamus*.

#### 5.1.2. Anti-Depressant Activity

In traditional Chinese medicine, *A. tatarinowii* is one of the most frequently employed anti-depressants. Kaixin San and Changpu Yujin decoctions are both traditional prescriptions for the treatment of neurological disorders in which *A. tatarinowii* plays a crucial role [86,87]. The tail suspension test for mice and the forced swimming test for rats are models of behavioral melancholy; it is well known that they can mimic the cause and certain symptoms of depression. The anti-depressant effects of the alcohol precipitate and water extract of *A. tatarinowii* were demonstrated via the tail suspension test with mice and the forced swimming test with rats by the authors of [88]. The anti-depressant properties of the alcohol precipitation extract surpassed those of the water extract. At concentrations of 2.5 g/kg, 5.0 g/kg, and 10.0 g/kg, the immobility time of forced swimming decreased by 8.3%, 2.4%, and 9.8%, respectively, relative to the water extraction group. The immobility time of tail suspension decreased by 9.7%, 14%, and 12.2%. We discovered that the effect of fluoxetine did not significantly differ from that of the alcohol extract group, as elucidated in [89]. The essential oil and asarone extracted from rhizomes produced anti-depressant-like properties. The essential oil (120 mg/kg), α-asarone (10 and 20 mg/kg), and β-asarone (20 mg/kg) demonstrated anti-depressant-like effects; they could substantially reduce an animal’s period of immobility. Blocking the reuptake of monoamine neurotransmitters such as 5-HT increases the amount of bioavailable 5-HT in the synaptic space of nerve cells, thereby acting as an anti-depressant. It has been proposed that the anti-depressant mechanism of *A. tatarinowii* may be related to a reduction in the content of the pro-inflammatory factors IL-1 and IL-6 and an increase in the content of the pro-inflammatory factors IL-10 and IL-13 in serum [90,91]. 

In another study, β-Asarone (25 mg/kg) influenced the expression of NSE in the hippocampus of rats with chronic mild unpredictable stress (*p* < 0.05) but had no significant effect on the NSE serum level of rats. β-Asarone may have relieved the depression of rats by improving the activity and reducing the damage of neurons, thereby exerting a protective effect on the nerves of depressed rats [92]. The flavonoid apigenin in *A. calamus* selectively binds to the central benzodiazepine receptor with high affinity; this has important anti-anxiety and anti-depression activities (Figure 8) [93].

#### 5.1.3. Protective Activity against Alzheimer’s Disease

Alzheimer’s disease is an irreversible and progressive neurodegenerative disease related to age. AD is associated with decreased acetylcholine levels. Acetylcholinesterase inhibitors are also used in the management of AD [94]. When searching for natural AChE inhibitors from medicinal plants, some researchers have noted that *Acorus* plants have memory-enhancing characteristics. The main active compounds α-asarone and β-asarone are the most promising candidates for eliciting cognitive improvements (Figure 8). In one study, through elevated plus-maze, passive avoidance, and avoidance active tests, it was observed that α-asarone (15 and 30 mg/kg) could significantly improve memory impairments induced by scopolamine in rats, reducing the brain acetylcholinesterase activity in the cortex, hippocampus, and striatum regions of amnesic rats (*p* < 0.05). There were no definite visible side effects of α-asarone, even at a higher dose (30 mg/kg) [95]. α-Asarone (at doses of 2.5 µg/mL and 7.5 µg/mL) could also protect primary hippocampal neurons from glutamate-induced neuronal excitotoxicity, reducing neuronal damage (*p* < 0.01 and *p* < 0.0001, respectively). In terms of morphological alterations, a greater number of shrinking cells with a loss of the synaptic reticular structure were reduced by α-asarone pretreatments. α-Asarone (7.5 µg/mL) reduced hippocampal Aβ_1-42_ levels (*p* < 0.01) by regulating GABAAR to alleviate hippocampal hyperactivity disorder [96]. 

Recent evidence suggests that autophagy might be involved in the pathogenesis of AD. Autophagy is a self-degradative process that plays a critical role in removing long-lived proteins and damaged organelles. In one particular study, β-Asarone (at doses of 7.5, 15, and 30 μg/mL) protected cells from Aβ_1-42_-induced cytotoxicity via the activation of the AKT–mTOR signaling pathway; this process occurred by promoting autophagy [97]. In another study, β-Asarone (36 μM) inhibited Aβ by promoting autophagy; it decreased APP, PS1, BACE1, and p62 and increased the expression of SYN1, BECN1, and LC3 proteins. These are closely related to the pathogenesis of AD [98].

Studies have demonstrated that the methanol extract of *A. calamus* (200 mg/mL) could effectively inhibit cholinesterase activity [99]. For example, in one study, a decoction mixture of *A. tatarinowii* and *Polygala tenuifolia* (1:1) significantly reduced the subacute aging of mice caused by D-galactose, stimulated the production of oxygen free radicals in the brain tissue and blood of dementia-model mice (*p* < 0.01), and accelerated the clearance of lipid peroxide, thus reducing the damage of oxygen free radicals to brain tissue and preventing brain-tissue atrophy [100]. The combined application of ginseng and *A. tatarinowii* improved the learning and memory ability of mice in water maze, passive avoidance, and step-down tests, decreasing the APP/PS1 double-transgenic mice hippocampus Aβ_1-42_ and AChE levels as well as LC3B and Beclin-1 expressions. The number of autophagosomes also decreased, which suggests that ginseng and *A. tatarinowii* could treat Alzheimer’s disease through the PI3K/AKT/mTOR autophagy pathway [101]. Due to the multi-component and multi-target characteristics of traditional Chinese medicine, the mechanism of action of a traditional Chinese medicine compound composed of *A. tatarinowii* cannot be single. *A. tatarinowii* and *Polygala tenuifolia* and ginseng and *A. tatarinowii* have all treated amnesia and other symptoms in the way of compatibility since ancient times. Whether the active ingredient changes after the combination of two drugs may be a topic of future research.

#### 5.1.4. Anti-Epileptic Activity

Epilepsy is a common disease of the central nervous system. Epilepsy can cause irreversible damage to the brain. It may be caused by an abnormal increase in brain blood flow, resulting in excessive glucose metabolism; the excessive production of lactic acid, mitochondrial superoxide, and hydroxyl free radicals; and changes in the function of albumen [102]. One research study demonstrated that the ethanol extract of *A. calamus* (200 mg/kg) had a positive therapeutic effect on a rat model (induced by ferric chloride), significantly reducing the wet-dog shake behavior, spike wave discharges, superoxide dismutase activity, and level of lipid peroxidation in the cerebral cortex, thus preventing the development of epilepsy induced by ferric chloride [103].

In another study, α-Asarone from *Acorus* reduced the cell damage of neurons in a refractory epilepsy cell model, inhibiting γ-aminobutyrate transaminase GABA-T activity and reducing GABA catabolism by upregulating the expression of glutamic acid decarboxylase GAD 67. GABA synthesis increased and GABA-mediated inhibition was enhanced, playing an anti-epileptic role [104,105]. In another study, α-Asarone (50 mg/kg) improved the epileptic behavior of pentetrazol-induced rats by regulating the equilibrium of amino acid neurotransmitters and suppressing cell membrane damage [106]. This may be attributed to α-asarone having electron-donating methoxy groups in the 3,4,5 position on the phenyl ring, thus increasing the anti-epileptic potency. Existing research has proven that α-asarone may have properties related to mutagenicity, genotoxicity, and teratogenicity (Figure 8) [107]. It is necessary to pay attention to medication contraindications and dosage during clinical treatments to balance the therapeutic effect and toxic side effects.

### 5.2. Anti-Inflammatory Activity

**Figure 9:**
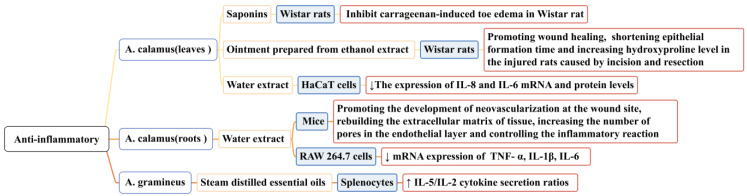
Anti-inflammatory activity of the *Acorus* genus.

When a host is stimulated by microorganisms or other detrimental substances, inflammation occurs. Under pathological conditions, inflammation can result in tissue destruction and even organ dysfunction. Long-term inflammation impedes wound tissue reconstruction and delays healing. The activation of inflammatory cytokines and chemokines and the recruitment of macrophages can promote wound healing. In the study of the anti-inflammatory properties of *Acorus*, the content of wound models and inflammatory factors have also been used to prove its anti-inflammatory activity [108,109]. Researchers have mainly studied the anti-inflammatory activities of *A. calamus* and *A. gramineus*; some conclusions related to this that have been reported in the literature are shown in Figure 9. In one study, saponins (at doses of 75, 150, and 300 mg/mL) isolated from the leaves of *A. calamus* significantly inhibited carrageenan-induced toe edemas in Wistar rats (*p* < 0.01) [110]. 

In another study, an ointment prepared from the ethanol extract of *A. calamus* leaves (40% *w*/*w* and 20% *w*/*w*) promoted wound healing and wound contraction, shortening the epithelial formation time and increasing the hydroxyproline level in injured rats [111]. In another study, a water extract from the roots of *A. calamus* (0.1/1/10 μg/mL) accelerated the healing and repair of incised wounds in mice, promoting the development of neovascularization at the wound site, rebuilding the extracellular matrix of the tissue, increasing the number of pores in the endothelial layer, and controlling the inflammatory reaction. It also effectively inhibited LPS-induced inflammation of rat macrophage RAW 264.7 and downregulated the mRNA expression of the following inflammatory factors: TNF-α, IL-1β, and IL-6 [76]. The water extract of *A. calamus* leaves (100 ng/mL) could effectively inhibit the production of pro-inflammatory cytokines IL-8 and IL-6 in HaCa T cells (*p* < 0.05). Its mechanism of action may be related to NF-κB activation and the phosphorylation of IRF3 [112]. 

In another study, the essential oil of *A. gramineus* (10 g/mL) significantly increased IL-5/IL-2 (Th2/Th1) cytokine secretion ratios via splenocytes (*p* < 0.05). *A. gramineus* has Th2 polarization characteristics and an anti-inflammatory potential [113]. The anti-inflammatory activity of the essential oil components of *A. calamus* has not yet been discovered, but this possibility cannot be ruled out. Current research on the specific anti-inflammatory process of *Acorus* is not detailed enough; the anti-inflammatory pathways and links should be studied further.

### 5.3. Anti-Bacterial Activity

The anti-bacterial effect of traditional Chinese medicine exerts an anti-infective effect at an overall level, with the characteristics of a definite curative effect, minor side effects, and less drug resistance. Obtaining anti-bacterial active substances from traditional Chinese medicine has a positive role in the development of anti-bacterial drugs [113,114,115].

**Figure 10:**
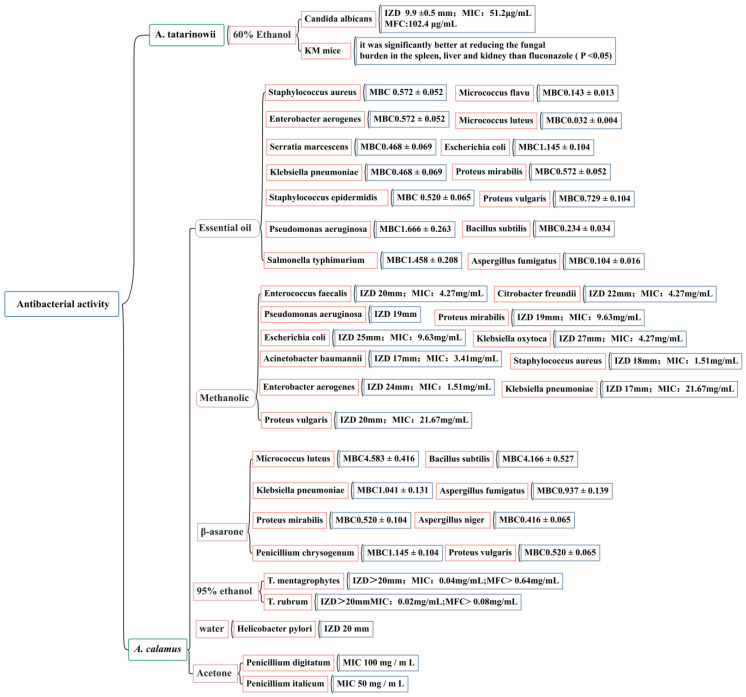
Anti-bacterial activity of *A. tatarinowii* and *A. calamus*.

At present, domestic and foreign research on the anti-bacterial activity of *Acorus* plants has mainly focused on the two species of *A. tatarinowii* and *A. calamus* by measuring the IZD, MIC, MBC, and MFC. The effects of the volatile oil; ethanol, methanol, acetone extracts; and β-asarone from *A. calamus* on 25 common pathogenic bacteria (including *Staphylococcus aureus*, *Staphylococcus epidermidis*, *Escherichia coli*, *Candida albicans*, *Helicobacter pylori*, and *Micrococcus luteus*) have been evaluated [116,117,118,119,120,121,122]. The accurate IZD, MIC, MBC, and MFC figures are shown in Figure 10. It has been noted that there is a wide range of endophytic bacteria with anti-bacterial activity in *A. calamus*, accounting for 40.7% of the total number of endophytic bacteria; highly active strains account for 12.39% of the total number of endophytic bacteria. This demonstrates that there are abundant endophytic resources in *A. calamus* and that its metabolites have potential anti-bacterial substances. It is necessary to further study the effective ingredients of *A. calamus* to clarify its anti-bacterial mechanism and ensure the safety and effectiveness of the development of *Acorus* anti-bacterial drugs.

### 5.4. Effect on Cardiovascular System

#### 5.4.1. Myocardial Cell Protection Activity

Coronary heart disease and acute myocardial infarction are the primary diseases that cause human deaths worldwide. The main method used to treat these diseases involve restoring myocardial blood and oxygen supply. However, supplying blood/oxygen causes severe myocardial tissue damage, namely, MIRI. MIRI can cause irreversible cardiac cell death and even lead to heart failure. The myocardium is the tissue with the highest oxygen uptake in the body; myocardial cells are particularly sensitive to various factors that can cause myocardial ischemia and hypoxia. If myocardial ischemia is damaged, it leads to cell membrane damage, myocardial enzyme leakage, and myocardial cell necrosis [123,124,125]. How to protect myocardial cells is a hot topic in the study of cardiovascular diseases. 

**Figure 11:**
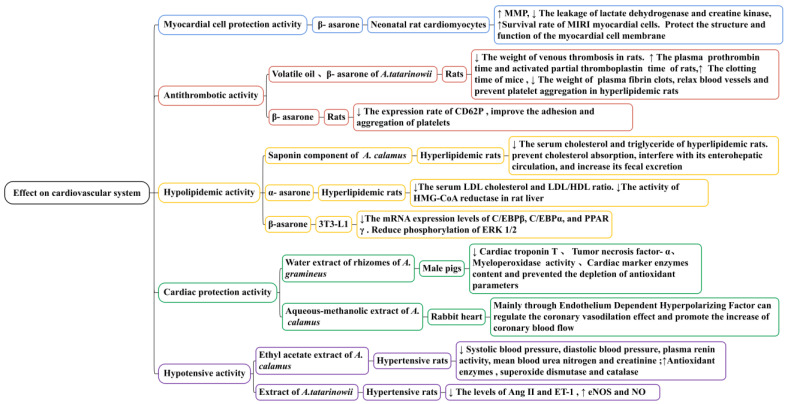
Protective effects for the cardiovascular system provided by the *Acorus* genus.

Through in vitro and in vivo experiments (Figure 11), it has been confirmed that β-asarone has a significant protective effect on MIRI myocardial cells. β-Asarone (at doses of 25, 12.5, and 6.25 μg/mL) could reduce the ischemia/reperfusion injury of neonatal rat cardiomyocytes induced by Na_2_S_2_O_4_ to different degrees; in one study, it increased MMP (*p* < 0.05), decreased the leakage of lactate dehydrogenase and creatine kinase, and protected the structure and function of myocardial cell membranes. *A. tatarinowii* volatile oil (75 mg/kg) and β-asarone (40 mg/kg) significantly reduced the level of endothelin (*p* < 0.05 and *p* < 0.01, respectively), increased the level of NO (*p* < 0.01 and *p* < 0.01, respectively), and reduced the degree of myocardial tissue damage and necrosis rate in rats with myocardial ischemia. Its mechanism may be related to reducing cell membrane damage and mitochondrial membrane permeability [126,127,128]. Whether it is necessary to pay attention to the dosage and whether there are other toxic or side effects when using β-asarone to treat myocardial cell injuries remain to be studied.

#### 5.4.2. Anti-Thrombotic Activity

Thrombosis is a disease in which the clotting system of the body is disrupted, leading to blood vessel blockage, the ischemia of organs, and dysfunction; an increase in fibrinogen and a decrease in prothrombin and thrombin time are the direct causes of thrombosis [129,130]. Studies have demonstrated that *A. tatarinowii* volatile oil (75 mg/kg) and β-asarone (40 mg/kg) could reduce the weight of venous thrombosis in rats (Figure 11). For example, in one study, the inhibition rates were 22% and 16%, respectively. It significantly prolonged the plasma prothrombin time (*p* < 0.01 and *p* < 0.001, respectively) and activated the partial thromboplastin time (*p* < 0.05 and *p* < 0.001, respectively) of rats. It improved the hemorheology of rats with hyperviscosity induced by high-molecular dextran, especially the whole blood low shear viscosity and plasma viscosity. It also significantly prolonged the clotting time of mice (*p* < 0.001), reduced the weight of plasma fibrin clots, and prevented platelet aggregation in hyperlipidemic rats [131]. Plasma CD_62P_ and CD_63_ can promote the formation of the thrombus. CD_62P_ is a platelet activation-dependent granule surface facial mask protein, and CD_63_ is a platelet lysosomal membrane protein component. Their levels reflect the activation of platelets and the degree of endothelial cell damage. In one study, β-Asarone (42.4 μg/g) significantly reduced the expression rate of CD_62P_ (*p* < 0.01) and improved the adhesion and aggregation of platelets. Therefore, it may have a role in preventing and treating thrombotic cerebrovascular disease [132].

#### 5.4.3. Hypolipidemic Activity

Hyperlipidemia is a metabolic disease and the most significant risk factor for cardiovascular and cerebrovascular diseases as well as metabolic diseases such as coronary heart disease, atherosclerosis, and obese livers. It is characterized by an increase in serum total cholesterol, triglyceride, and LDL levels, whereas HDL levels decrease [133,134,135]. Recently, studies have confirmed that the saponins of *A. calamus* and asarone have a hypolipidemic effect (Figure 11). A saponin component (10 mg/kg) isolated from the alcohol extract of *A. calamus* significantly reduced the serum cholesterol and triglyceride of hyperlipidemic rats (*p* < 0.01). This saponin component could prevent cholesterol absorption, interfering with its enterohepatic circulation and increasing its fecal excretion [136]. 

In one specific study, α-Asarone (80 mg/kg) decreased the serum total cholesterol by approximately 40% in hypercholesterolemic rats. It affected the level of serum low-density lipoprotein cholesterol, inhibited the activity of HMG-CoA reductase in the liver, and significantly increased the bile flow ratio. These effects of α-asarone were consistent with those previously obtained with other HMG-CoA reductase inhibitors, such as lovastatin and simvastatin [137]. In another study, β-Asarone (at doses of 0.03, 0.06, 0.13, and 0.25 mM) significantly inhibited intracellular lipid accumulation during 3T3-L1 adipocyte differentiation in a concentration-dependent manner. In the differentiation process in 3T3-L1 cells treated with β-asarone, the protein and mRNA expression levels of C/EBPβ, C/EBPα, and PPARγ decreased, and the phosphorylation of ERK1/2 was reduced. The results of this study demonstrated that β-asarone could reduce lipids by inhibiting the expression of adipogenic transcription factors; it has the ability to suppress adipogenesis and inhibit PPARc expression [45].

#### 5.4.4. Cardiac Protection Activity

Currently, there is limited research on the heart protection effects of *Acorus* plants (Figure 11). The existing research mainly focuses on *A. gramineus* and *A. calamus*. In one study, water extracts of the rhizomes of *A. gramineus* (50 and 100 mg/kg) significantly improved isoproterenol-induced cardiac dysfunction in male pigs, reducing the cardiac troponin T, tumor necrosis factor-α, and cardiac marker enzyme contents, as well as the myeloperoxidase activity. This demonstrates that the water extract of the rhizomes of *A. gramineus* has significant cardiac protection potential and may be used to treat and prevent myocardial infarction [138]. The aqueous–methanolic extract of *A. calamus* has a therapeutic effect on ischemic heart disease mainly through the endothelium-dependent hyperpolarizing factor, which can regulate the coronary vasodilation effect and promote an increase in coronary blood flow [139].

#### 5.4.5. Hypotensive Activity

Hypertension is a common cardiovascular disease characterized by elevated blood pressure in systemic circulation arteries that can damage the structure and function of the heart, brain, kidneys, and other organs. Studies have demonstrated that the ethyl acetate extract of the *A. calamus* rhizome (250 mg/kg) could significantly decrease systolic blood pressure, diastolic blood pressure, plasma renin activity, and mean blood urea nitrogen and creatinine (*p* < 0.01, *p* < 0.01, *p* < 0.05, *p* < 0.01, and *p* < 0.01, respectively) in hypertensive rats induced by arterial clamps, significantly increasing anti-oxidant enzymes such as glutathione, superoxide dismutase, and catalase (*p* < 0.05, *p* < 0.01, and *p* < 0.001, respectively) and improving closed hypertension caused by renal artery occlusions in rats [140]. A reduction in NO synthesis reduces the responsiveness of endothelium-dependent vasodilators to blood vessels, weakening the vasodilator function and accelerating the formation and development of hypertension. NO is the synthesis of NOS with L-arginine as the substrate. eNOS is produced by vascular endothelial cells; its overexpression causes the activity level of eNOS and NO to be upregulated. In one specific study, *A. tatarinowii* extract (at doses of 45, 30, and 15 mg/kg) reduced the levels of Ang II and ET-1 in rats with essential hypertension (*p* < 0.05). After 3 weeks of administration, it significantly reduced blood pressure in rats (*p* < 0.05). Although the levels of eNOS and NO increased at different degrees, it could increase the synthesis of NO by activating the eNOS pathway of the vascular endothelium, thus causing vasodilation. It had a certain therapeutic effect on hypertensive rats with myocardial hypertrophy (Figure 11) [141].

### 5.5. Anti-Tumor Activity

Cancer has become one of the most serious diseases threatening human life. Its occurrence and development are complex pathological processes that involve multiple factors, multiple gene changes, and multiple stages of development. Modern medicine can be used to treat different cancers by means of radiotherapy and chemotherapy; however, this sometimes brings serious toxicity, drug resistance, and a risk of recurrence. The intervention of traditional Chinese medicine treatments can significantly reduce adverse reactions in the process of tumor treatments, improving the treatment efficiency and quality of life of tumor patients. The anti-tumor effect of traditional Chinese medicine has received greater attention and recognition. The anti-tumor activity and mechanism of traditional Chinese medicine have the characteristics of multiple targets and multiple pathways, such as those pertaining to tumor cell proliferation inhibition, tumor cell apoptosis promotion, tumor cell invasion inhibition, tumor microenvironment regulation, anti-tumor angiogenesis, and drug resistance reversal [142,143].

**Figure 12:**
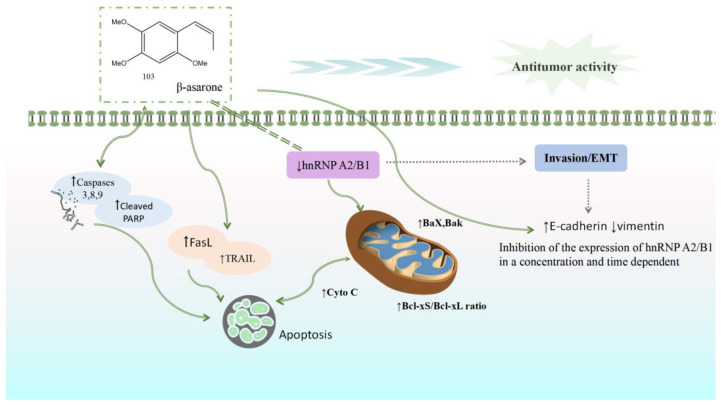
β-Asarone can increase the ratio of Bcl-xS/Bcl-xL by inhibiting the hnRNPA 2/B1 signal pathway, activating the death receptor proteins TRAIL and FasL, increasing the expression of cleaved caspases 3, 8, and 9, and inducing cell apoptosis. β-asarone can also inhibit the hnRNP A2/B1-mediated signal pathway by upregulating the expression of EMT, suggesting that it may block the epithelial–mesenchymal transformation of cancer cells.

Current research on the anti-cancer activity of *Acorus* plants has primarily concentrated on glioblastoma, colon cancer, gastric cancer, and prostate cancer, as shown in Table 1. Extracts of *A. calamus* and β-asarone can play an anti-tumor role by inhibiting the proliferation and invasion of tumor cells, promoting cell apoptosis, inhibiting tumor angiogenesis, and facilitating cell cycle arrest. β-Asarone may induce the apoptosis of U251, LoVo, HT29, and SW480 cells by inhibiting the hnRNP A2/B1 signal pathway. hnRNP A2 and B1 are two structural homologous proteins belonging to the hnRNP protein family; these are widely expressed in most human tissues and have important functions. In recent years, studies have observed that hnRNPA2/B1 has a differential expression in various malignant tumors and that it can participate in regulating gene expressions, promoting proliferation and migration, inhibiting apoptosis, and regulating tumor drug resistance. β-Asarone increases the Bcl-xS/Bcl-xL ratio by regulating the level of key proteins in cell death and the mitochondrial apoptosis pathway. With the activation of death receptor protein TRAIL and FasL, the expression of cleaved caspases 3, 8, and 9 increases, leading to the induction of apoptosis. β-Asarone downregulates the expression of vimentin or N-cadherin by upregulating the expression of EMT, inhibiting the signal pathway mediated by hnRNP A2/B1, and reducing the expression of EMT. This suggests that β-asarone may block the EMT process of cancer cells (Figure 12).

### 5.6. Effect on Digestive System

The rhizome of *A. calamus* is widely used as an emetic and stomach-nourishing medicine to treat indigestion, gastric colic, childhood dysentery, and diarrhea. By taking 1–3 g or 2–7 g of the rhizome of *A. calamus* and decocting it, it can be used to treat abdominal pain and diarrhea [150]. Modern pharmacological studies have demonstrated that the methanol extract (15 mg/kg) and water extract (15 mg/kg) of *A. calamus* has a significant anti-diarrheal effect (*p* < 0.001) on a mouse diarrhea model caused by castor oil, which can be mediated by reducing the activity of Na + K + ATPase in the small intestine [151]. The ethanol extract of *A. calamus* (500 mg/kg) has been shown to reduce gastric ulcers in mice (caused by pylorus ligation by taking indomethacin, reserpine, and cysteamine), demonstrating significant anti-secretory and anti-ulcer activities [152]. These studies confirmed that *A. calamus* may become a common drug for gastrointestinal disorders (such as acute abdominal pain and bleeding). Its long-term clinical safety requires further study.

**Figure 13:**
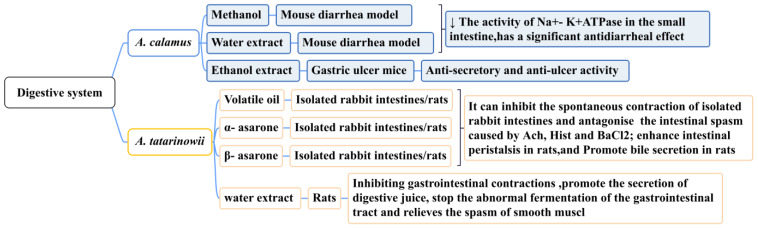
The effect of the *Acorus* Genus on the digestive system.

Traditional Chinese medicine believes that *A. tatarinowii* enters the stomach meridian and fulfills the function of dissolving dampness and harmonizing the stomach. Studies have demonstrated that it can relax the gastric smooth muscle, inhibit intestinal spasms, and promote gastrointestinal peristalsis. In one study, the total volatile oil, α-asarone, and β-asarone of *A. tatarinowii* were shown to inhibit the spontaneous contraction of isolated rabbit intestines and antagonize intestinal spasms caused by acetylcholine, histamine diphosphate, and BaCl_2_. The lowest effective concentrations were 2.0 mg/mL, 5.3 μg/mL, and 7.32 μg/mL. These ingredients also enhanced intestinal and bile secretion in rats [153]. Gastrointestinal myoelectric activity is one of the sensitive indicators used to study the form and regularity of gastrointestinal movements. Research has demonstrated that *A. tatarinowii* has different degrees of inhibition on the frequency and amplitude of gastric and duodenal spikes and slow waves, as well as the occurrence rate of duodenal spikes. The water extract of *A. tatarinowii* (4 g/kg) could significantly inhibit the slow-wave frequency (*p* < 0.01) and peak amplitude (*p* < 0.01) of gastric electricity in rats [154]. The water extract of *A. tatarinowii* could also promote the secretion of digestive juices, prevent the abnormal fermentation of the gastrointestinal tract, and relieve smooth muscle spasms (Figure 13) [155]. 

### 5.7. Anti-Diabetes Activity

**Figure 14:**
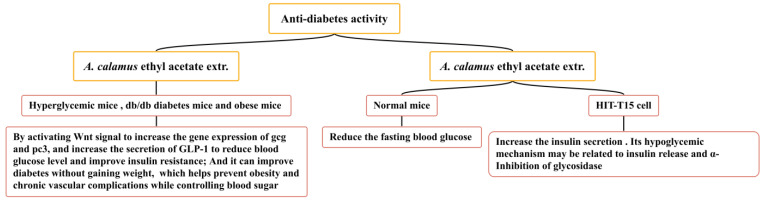
Anti-diabetes effect of *A. calamus*.

Diabetes mellitus is an endocrine and metabolic disease caused by defects in pancreatic secretion or insulin function. Based on the high acceptance of natural plant ingredients by patients, the hypoglycemic activity of natural products has become the focus of current medical research [156]. *A. calamus* is widely used in the traditional folk medicine of America and Merak (Banten, Indonesia) to improve diabetes. Several studies have confirmed that the ethyl acetate extract of *A. calamus* has an anti-diabetes effect (Figure 14), increasing the secretion of GLP-1 and inhibiting α-glycosidase activity, which can reduce blood sugar. *A. calamus* ethyl acetate extract (100 mg/kg) has been shown to exert a hypoglycemic effect on hyperglycemic mice (induced by STZ), db/db diabetes mice, and obese mice (induced by a high-fat diet). It may increase the gene expression of gcg and pc3 by activating the Wnt signal and reducing the blood glucose level by directly or indirectly increasing the secretion of GLP-1 [157]. The ethyl acetate extract of *A. calamus* has been shown to promote the differentiation of 3T3-L1 preadipocytes to a certain extent; it has also been shown to have potential insulin-sensitizing activity and release the α-glucosidase inhibition mechanism. It has a hypoglycemic role and does not increase weight, thus improving diabetes and helping to prevent obesity and chronic vascular complications whilst controlling blood sugar [158,159]. In one particular study, the ethyl acetate extract of *A. calamus* (400 and 800 mg/kg) significantly reduced the fasting blood glucose of normal mice and increased the insulin secretion of HIT-T15 cells. Its hypoglycemic mechanism may be related to insulin release and the α-inhibition of glycosidase [160].

## 6. Clinical Application

The monomer compounds, extracts, and volatile oils extracted and separated from *Acorus* have been widely used in clinical practice.

### 6.1. Respiratory System

Asarone and caryophyllene, the components of *Acorus*, have a positive effect on inhibiting responses to acute asthma and can treat all types of chronic bronchitis [161]. Patients with severe bronchial asthma in a clinical trial chewed the fresh powder of *A. calamus* for 2–4 weeks; it was observed that it had a positive anti-asthmatic effect, and no side effects were recorded. Asthmatic patients also significantly relieved bronchospasms by chewing small pieces of *A. calamus* [162]. 

### 6.2. Nervous System

Currently, the effective rate of treating epilepsy with asarone is 83%. *A. tatarinowii* is widely used in the treatment of closed syndrome and faint syndrome; its clinical effect is extremely accurate. Clinically, an injection (0.5% total volatile oil solution) comprising the single volatile oil of *A. tatarinowii* was used to treat pulmonary encephalopathy comas with an effective rate of 74.97%; it rapidly eliminated disturbances of consciousness and neuropsychiatric symptoms [163]. 

*A. calamus* has anti-convulsant activity. A combination of a water extract and an alcohol precipitate with carbamazepine had a stronger protective effect on the nervous system and was an effective anti-epileptic drug [164]. *A. calamus* has positive effects on insomnia, depression, neurasthenia, epilepsy, and amnesia. It is mainly used to treat epilepsy, fever, dizziness, wet phlegm, forgetfulness, and drowsiness. Indian herbal medicine uses it to treat various neurological and mental disorders, including epilepsy, mental disorders, memory loss, insomnia, depression, and neurasthenia [165]. 

### 6.3. Digestive System

*A. calamus* has the effect of removing dampness and strengthening the stomach. It can be used to treat vomiting, diarrhea, gastrointestinal flatulence, malaria, inflammation, dysentery, and other symptoms [166]. 

### 6.4. Cardiovascular System

The crude extract of *A. calamus* has the effect of dilating coronary vessels, which provides a basis for the treatment of ischemic heart disease [139]. The essential oil of *A. calamus* has the effect of anti-atrial fibrillation, atrial fibrillation, ventricular arrhythmia, etc. Clinically, it has been observed that the health status of patients with ischemic heart disease significantly improved after the use of *A. calamus* [167].

### 6.5. Urinary System

*A. calamus* has been employed as a mild diuretic, improving the quality of urine. A combination with milk is useful in obstructive urinary disorders, particularly for distended urinary bladders [168].

## 7. Application in Water Pollution Control

Due to their fast growth, large biomass, and strong absorption capacity, *Acorus* plants are favored in the application of eutrophic water treatments. As an important constructed wetland plant, *Acorus* can absorb N, P, heavy metals, and other pollutants in water and has an important ecological and environmental value. At present, the *Acorus* plants used for water pollution control mainly include *A. calamus* and *A. tatarinowii*. In the secondary river area of the Three Gorges in China, these two plants are used as the main floating island plants to purify the eutrophic water, yielding positive results [169,170].

In one study, it was identified that the removal rates of total nitrogen, total phosphorus, chloride, and NH_4_-N by *A. tatarinowii* were 84.7%, 43.9%, 89.5%, and 89.5%, respectively. The DO (dissolved oxygen) increased by 26.6%. The removal rates of TN, TP, and NH_3_-N by *A. tatarinowii* were 53.99%, 55.6%, and 50.76%, respectively. The COD (chemical oxygen demand) decreased by 48.55% [9]. The removal of TN and TP by *A. calamus* in a water body increased with the increase in the initial concentration in the water body, and the growth status of a water body with high- and medium-concentration pollution was better than that of a sewage body with a low concentration [171,172]. *Acorus* can control water eutrophication by inhibiting the specific growth of algae. *A. tatarinowii* and *A. calamus* have significant inhibitory effects on algae such as *Chlorella vulgaris*, *Scenedesmus obliquus*, *Anabaena floatuae*, *Selenastrum carpricornutum*, *and Microcystis aeruginosa. Acorus* releases allelochemicals and fat-soluble organics through its root exudates to produce an anti-algae effect. The strength of the effect depends on the relative biomass between *Acorus* and the algae [173,174]. To a certain extent, *Acorus* is also considered to be an indicator plant for water body cleaning.

Heavy metal pollution in water environments is an important aspect of water pollution, causing significant harm to aquatic plants and animals. Phytoremediation technology has paved the way for ecologically friendly and economically valuable methods for the treatment of heavy metal pollution in water and can be widely used. Generally, aquatic plants with a high tolerance to high concentrations of heavy metals are used to absorb, transform, filter, and fix heavy metal ions in water to reduce the bioavailability of heavy metal pollutants [175,176,177,178]. In one study, *A. calamus* effectively absorbed V^5+^, Cr^6+^, and Cd^2+^ from water, yielding the highest removal rates of 52.4%, 46.8%, and 90.0%, respectively [179]. Sb^3+^ and Sb^5+^ caused unfavorable effects on the growth of *A. calamus*, but the seedlings did not die and were modestly adaptive and Sb-accumulative. This suggests that *A. calamus* may be a candidate for phytoremediation in Sb-polluted areas [180]. The roots and stems of *A. tatarinowii* have a strong ability to enrich heavy metals such as Ni, Cu, Pb, Cr, As, and Zn in sewage; in one study, the rooting ability of plants in sewage was enhanced, the root system increased, and the root activity was significantly enhanced compared with those in tap water [181,182].

*Acorus* plants are widely distributed in the world and are rich in resources. Research on the treatment of sewage using *Acorus* has significant practical value. *Acorus* has demonstrated both economic value and environmental value. *Acorus* in small water bodies such as streams and ponds can reduce pollution at the source and continuously remove the nutrient elements in the water body through harvesting as a medicinal material. *Acorus* plants are expected to become the first plants to be selected for improvements to eutrophic water and to treat heavy-metal-polluted water.

## 8. Discussion

The ethnic, botanic, phytochemistry, pharmacological, and clinical applications of *Acorus* plants, as well as their role in water pollution control, have been systematically reviewed in this paper. Currently, 224 compounds have been isolated from *Acorus*; volatile components form the majority of these compounds, and volatile oil is the main pharmacologically active component. Modern pharmacological research indicates that *Acorus* plants have neuroprotective, anti-depressant, anti-epileptic, anti-diabetic, anti-inflammatory, anti-hypertensive, and gastric colic effects. Clinical studies have observed that *Acorus* plants have broad application prospects in areas such as bronchial asthma, neurasthenia, epilepsy, cardiovascular protection, and gastrointestinal inflammation. *Acorus* is also widely used in traditional Chinese medicine prescriptions, and using it in combination with other herbs can yield improved medical effects. As an important artificial wetland plant, *Acorus* is widely used to absorb pollutants such as N, P, and heavy metals in water; it is an important species when treating water pollution. Overall, *Acorus* has significant medicinal and environmental value and is worthy of further research.

To better develop and apply *Acorus*, the following issues must be addressed: The chemical composition of *Acorus* is relatively complex. Among the numerous components found in *Acorus* plants, the volatile oil component is the main pharmacologically active component. Under different harvesting times and storage conditions, the content of the volatile components in medicinal materials varies; the quality and efficacy of the medicinal materials may also change. To ensure its efficacy, in-depth research on the harvesting period and storage conditions of *Acorus* plants is required.

The pharmacological research regarding *Acorus* plants mainly focuses on diseases related to the nervous system. α-Asarone and β-asarone are the material basis for the clear pharmacological effects of *Acorus* and are widely used in clinical practice. α-Asarone and β-asarone exhibit different pharmacological activities at different doses and have demonstrated a wide range of pharmacological activities at lower doses (<50 mg/kg), including anti-depressant, anti-anxiety, anti-Alzheimer’s disease, and anti-Parkinson’s disease activities. At higher doses (≥50 mg/kg), they have demonstrated low motility, impaired motor coordination, hypothermia, anti-epilepsy, anticancer, anti-hyperlipidemia, anti-thrombotic, anti-cholestasis, and radiation protection activities [106]. It is necessary to strengthen the research on the dose–effect relationship of α-asarone and β-asarone to ensure a more accurate representation of *Acorus* plants in clinical applications.

*Acorus* is a type of traditional Chinese aromatic resuscitation medicine. It contains volatile components closely related to inducing resuscitation and restoring consciousness; it can improve the permeability of the blood–brain barrier. Further exploring the relationship between resuscitation drugs and volatile chemical components and methods for optimizing their utilization using modern preparation technology is of significance for the treatment of brain diseases.

*Acorus* is widely distributed all over the world; it is rich in resources and not only diverse in biological activity but also helpful for the restoration of eutrophic water. In China, ethnic minorities currently use *Acorus* as seasoning to remove food odors and to brew *Acorus* wine. The aromatic oil extracted from *Acorus* can be used as a cosmetic and soap essence after refinement. Whether *Acorus* can be used in food research, as well as its other potential developments, in the future is an interesting research direction.

## Figures and Tables

**Table 1:** Anti-cancer pharmacological activity and mechanism of β-asarone.

Extract/ Compound	Animal/Cell Line	Mechanism/Results	Ref.
β-asarone (240 and 360 μM)	U251 cells	↓ expression of hnRNP H1, hnRNPA2/B1, and cathepsin D	[144]
β-asarone (30 and 60 μM)	U251 cells	Inhibition of cell migration, invasion, and adhesion; EMT via the upregulation of E-cadherin and downregulation of vimentin. Inhibition of the expression of hnRNP A2/B1 in a concentration and time-dependent way.	[145]
β-asarone (60, 120, 240 and 480 μM)	U251 cells; glioma U251 tumor xenograft model (nude mice)	↓ expression of matrix metalloproteinases (MMP-9) and p-STAT3.Cell cycle arrest at G1 phase, ↓ expression of matrix metalloproteinases (MMP-9) and p-STAT3. ↑ Bcl-xS/Bcl-xL, the inhibition of hnRNP A2/B1-mediated signaling pathway. Activation of the death receptor proteins TRAIL and FasL. Cleavage of caspase 8 and caspase 3.	[146]
β-asarone (200 and 400μM)	LoVo cells	Induced cell apoptosis via annexin V↓ mitochondrial membrane potential (MMP). ↑ proapoptotic proteins (Bax expression), and ↓ anti-apoptotic regulators Bcl-2, Bcl-xL	[147]
β-asarone (0, 10, 30 and 100 nM) (50, 100 and 200 μg/kg)	HT29 and SW480 cells; 1, 2-dimethyl hydrazine induced colorectal cancer mice	↓ Bcl-2/Bax and ↑ the executer apoptosis enzyme caspase-9 and caspase-3 cascades. Induces cell senescence (SA-β-Gal activity). ↑in the levels of lamin B1, as well as p53, p21, and p15. Reduction in the incidence and number of formed tumors. Induction of senescence in human colorectal cancer via the activation of lamin B1 by promoting the tumor protein (p53, p21) expressions.	[148]
Ethanolic extracts of *A. calamus* (15, 30, 60, 120, 240 and 480 μg/mL)	AGS cells	Inhibit cell proliferation, cell cycle arrest at G1 phase. Inhibition of formation of tube-like structures confirming anti-angiogenic properties.	[149]
Ethanolic extracts of *A. calamus* (250, 500 and 750 μg//mL)	LNCaP cells	Inhibition of cell viability. Induces apoptotic cell death.	[36]

↑, up-regulation or increase; ↓, inhibition, alleviation or reduction.

## Data Availability

No data were used for the research described in the article.

## Sample Availability

Not available.
